# Nearly Complete Genome Sequence of Brugia pahangi FR3

**DOI:** 10.1128/MRA.00479-20

**Published:** 2020-07-02

**Authors:** John Mattick, Silvia Libro, Benjamin C. Sparklin, Matthew Chung, Robin E. Bromley, Suvarna Nadendla, Xuechu Zhao, Sandra Ott, Lisa Sadzewicz, Luke J. Tallon, Michelle L. Michalski, Jeremy M. Foster, Julie C. Dunning Hotopp

**Affiliations:** a Institute for Genome Science, University of Maryland School of Medicine, Baltimore, Maryland, USA; b Division of Protein Expression and Modification, New England Biolabs, Ipswich, Massachusetts, USA; c Department of Biology and Microbiology, University of Wisconsin Oshkosh, Oshkosh, Wisconsin, USA; d Department of Microbiology and Immunology, University of Maryland School of Medicine, Baltimore, Maryland, USA; e Greenebaum Cancer Center, University of Maryland School of Medicine, Baltimore, Maryland, USA; Broad Institute

## Abstract

Brugia pahangi is a zoonotic parasite that is closely related to human-infecting filarial nematodes. Here, we report the nearly complete genome of Brugia pahangi, including assemblies of four autosomes and an X chromosome, with only seven gaps. The Y chromosome is still not completely assembled.

## ANNOUNCEMENT

We sequenced the zoonotic parasitic roundworm Brugia pahangi FR3, which was originally obtained from a green leaf monkey in Kuala Lumpur and is distributed by the NIAID Filariasis Research Reagent Resource Center, known as FR3 (1). The 107,643,863 Illumina HiSeq 2500 paired-end 150-bp reads were generated from KAPA Hyper libraries using Brugia pahangi FR3 genomic DNA acquired from BEI Resources. Genomic DNA for PacBio RS II sequencing was obtained from 16 adult male and 16 adult female frozen worms obtained from FR3, which were homogenized in Qiagen G2 buffer with RNase A, and purified using Qiagen gravity-flow Genomic-tips with 80 U of proteinase K and DNA precipitation by centrifugation in the presence of 20 μg of glycogen. PacBio RS II data (P6C4 chemistry; 3,267,281 reads; average read length, 8,695 bp; *N*_50_, 25.4 kbp; maximum read length, 139 kbp) were generated from Sage Blue Pippin size-selected (>15 kbp) SMRTbell v1.0 libraries constructed using Covaris gTUBE-fragmented DNA. For Oxford Nanopore Technologies (ONT) sequencing, 185 adult female worms acquired from TRS Labs (Athens, GA, USA) were ground in liquid nitrogen, and DNA was extracted with a single phenol-chloroform DNA extraction, with spooling from an ethanol precipitation. Rapid libraries were constructed (SQK-RAD004) four times, from 4 μg, 1.8 μg, 0.9 μg, and 0.2 μg DNA. The latter three libraries were sequenced with an R9.4 MinION flow cell (FLO-MIN106), replacing the loading beads with water. The 4-μg library was modified using 1.5 μl of DNA fragmentation mixture and 3.5 μl of 10 mM Tris-Cl (pH 8.0) and 0.02% Triton X-100 (2) and was sequenced with a different R9.4 MinION flow cell, compared with the previous runs. This collectively resulted in 727,358 reads, which were called using Guppy v3.1.5 (average read length, 10,109 bp; *N*_50_, 22 kbp; maximum read length, 828 kbp).

**FIG 1:**
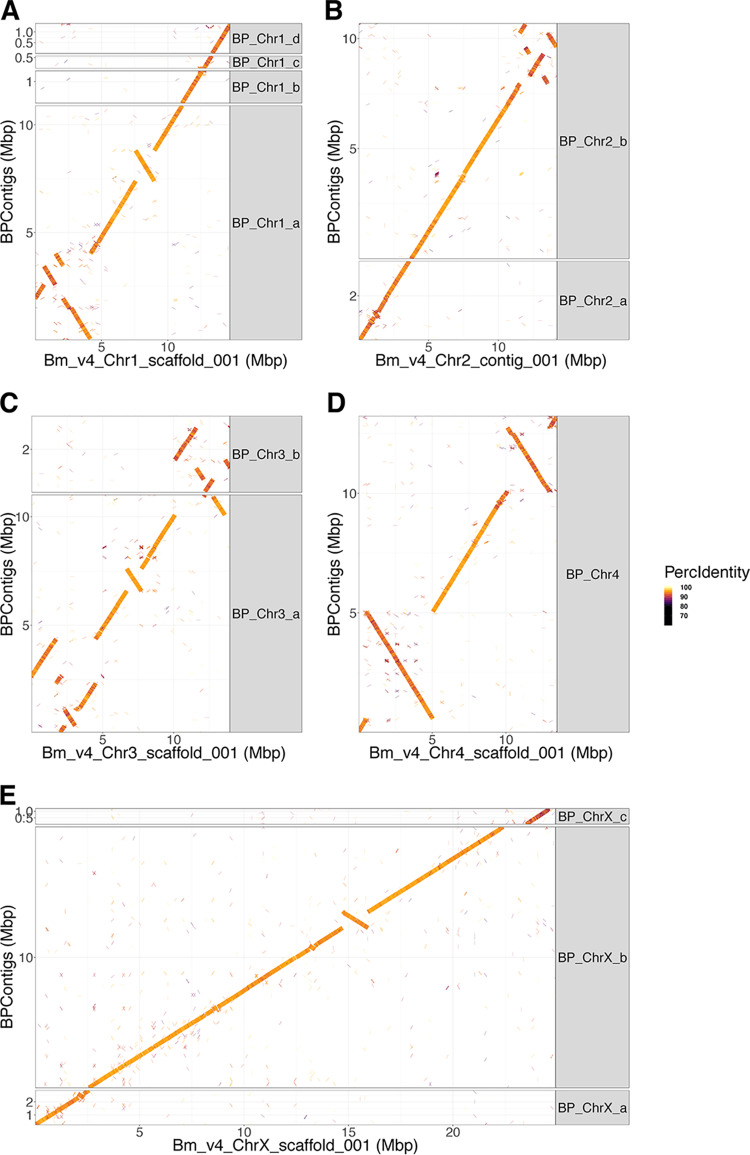
Synteny between Brugia pahangi and Brugia malayi chromosomes. The current Brugia pahangi genome was aligned to the Brugia malayi V4 genome (7) using NUCmer v3.1 (default options). The resulting delta file was converted into tabular form using the show-coords function with the option -qlTHb. Contigs >4.5% of the length of the matching B. malayi chromosome were identified in R (v. 3.4.4 [9]; libraries: RColorBrewer, ggplot2, data.table) and plotted with ggplots by chromosome. (A) Chromosome 1; (B) chromosome 2; (C) chromosome 3; (D) chromosome 4; (E) chromosome X. This nearly complete *B. pahangi* genome spans most of the B. malayi genome with some rearrangements. The orthologous regions of the genomes vary in percent identity but typically show >90% identity, with more nucleotide variation at the ends of chromosomes, relative to the middle of the same chromosomes, using the B. malayi chromosomes as references.

Illumina reads after quality control and trimming with FastQC v0.11.7 (3) and Trimmomatic v0.38 (4), respectively, and untrimmed PacBio and MinION reads were assembled with Canu v1.8 (5) multiple times but using different subsets of MinION experiments (excluding and including the 4-μg sample) and varying the estimated genome size as 90 Mbp and 100 Mbp. Default parameters were used except as otherwise noted. These assemblies were manually examined, including unique joins leading to a manual merger of assemblies. This manually merged assembly was indel corrected using Illumina reads and Pilon v1.22 (mindepth, 5; K, 85; minmq, 0; minqual, 35; fix indels) (6). Contigs were ordered and oriented according to the Brugia malayi reference genome (7), using NUCmer v3.1 to identify matches between the two genomes and to order B. pahangi contigs based on their B. malayi counterparts (Fig. 1). The genome spans 80.8 Mbp with seven gaps (*N*_50_, 11.2 Mbp; *L*_50_, 3), has a GC content of 29% with four autosomes and an X chromosome, and was deemed 96.4% complete with BUSCO v3 (8), with 206× Illumina, 182× PacBio, and 72× ONT sequencing depths. The remaining 142 contigs, spanning 16 Mbp, likely contain the mitochondrial genome, haplotypes, and pseudoautosomal and Y chromosome fragments.

### Data availability.

This genome has been deposited in GenBank under accession number JAAVKF000000000. The version described in this paper is the ﬁrst version, JAAVKF010000000. The raw data have been deposited in the SRA under the accession numbers SRX4135331, SRX4135330, SRX4135329, SRX4135328, SRX4135327, SRX4135326, SRX4135325, SRX4135324, SRX4135323, SRX4135322, SRX4135321, SRX4135320, SRX4135319, SRX4135318, SRX4135317, SRX4135316, SRX4135315, SRX4135314, SRX4135313, and SRX4135312 (PacBio), SRX7658407, SRX7658383, SRX7658378, SRX7658377, SRX7658352, SRX7658349, SRX7658341, SRX7658327, SRX7658323, SRX7658322, SRX7658317, and SRR10997235 (Illumina), and SRR11565851, SRR11472020, SRR11565849, and SRR11565826 (MinION).
